# Bone metastases from differentiated thyroid carcinoma: current knowledge and open issues

**DOI:** 10.1007/s40618-020-01374-7

**Published:** 2020-08-03

**Authors:** A. Nervo, A. Ragni, F. Retta, M. Gallo, A. Piovesan, V. Liberini, M. Gatti, U. Ricardi, D. Deandreis, E. Arvat

**Affiliations:** 1 Oncological Endocrinology Unit, Department of Medical Sciences, Città della Salute e della Scienza Hospital, University of Turin, Turin, Italy; 2 Nuclear Medicine, Department of Medical Sciences, Città della Salute e della Scienza Hospital, University of Turin, Turin, Italy; 3 Radiology Unit, Department of Surgical Sciences, Città della Salute e della Scienza Hospital, University of Turin, Turin, Italy; 4 Radiation Oncology, Department of Oncology, Città della Salute e della Scienza Hospital, University of Turin, Turin, Italy

**Keywords:** Thyroid cancer, Skeletal-related event, Metastatic disease, Cancer management

## Abstract

Bone represents the second most common site of distant metastases in differentiated thyroid cancer (DTC). The clinical course of DTC patients with bone metastases (BM) is quite heterogeneous, but generally associated with low survival rates. Skeletal-related events might be a serious complication of BM, resulting in high morbidity and impaired quality of life. To achieve disease control and symptoms relief, multimodal treatment is generally required: radioiodine therapy, local procedures—including surgery, radiotherapy and percutaneous techniques—and systemic therapies, such as kinase inhibitors and antiresorptive drugs. The management of DTC with BM is challenging: a careful evaluation and a personalized approach are essential to improve patients’ outcomes. To date, prospective studies focusing on the main clinical aspects of DTC with BM are scarce; available analyses mainly include cohorts assembled over multiple decades, small samples sizes and data about BM not always separated from those regarding other distant metastases. The aim of this review is to summarize the most recent evidences and the unsolved questions regarding BM in DTC, analyzing several key issues: pathophysiology, prognostic factors, role of anatomic and functional imaging, and clinical management.

## Introduction

Although differentiated thyroid carcinoma (DTC) accounts for only 3% of all reported malignancies [1], it is one of the five types of cancer that most frequently cause bone metastases (BM) [2]. In DTC population, BM occurs in 2–13% of all cases and can be detected in nearly half of the patients with distant metastases. After the lung, the skeleton is the second most common site of distant metastases in DTC [3, 4]. BM from DTC are generally osteolytic lesions with secondary bone formation in response to bone destruction and soft tissue involvement [4].

Any type of DTC can metastasize to bone structures. The rate of BM is threefold higher for follicular thyroid cancer (FTC, 7–28%) compared with papillary thyroid cancer (PTC, 1–7%) [5]. A likely explanation is that FTC more easily spreads via the blood stream to distant organs, due to a major tendency to invade blood vessels [6].

Axial skeleton, especially the spine and the pelvis, is the most common involved site. Cancer cells easily reach the red marrow of these bone segments since blood inflow is notoriously high. Furthermore, a preferential link between the thyroid gland and the axial skeleton is provided by Batson’s vertebral–venous plexus, which plays a role in the drainage of the head and neck region by indirect connections with the inferior thyroid veins [7].

Skeletal-related events (SREs), which include pathologic fractures, spinal cord compression, need for bone irradiation or surgery and malignant hypercalcemia, might be a serious complication of BM in DTC patients, resulting in quality of life impairment and high morbidity. Farooki et al*.* reported a 78% occurrence of SREs in DTC patients with BM; after a median of 10.7 months*,* 65% of them sustained a second SRE [8].

Prognosis of DTC patients with BM is generally poor and survival rates are lower than those observed with localisations at other distant sites [3, 9]: the overall survival (OS) at 10 years ranges from 13 to 21% [4].

## Pathophysiology

In recent years, the mechanisms underlying the development of BM from different types of cancer have been widely investigated.

The tumour-induced disregulation of the RANK–RANK ligand–osteoprotegerin pathway seems to be common to all osteolytic malignancies: activation of RANK leads to recruitment and maturation of osteoclasts, which directly influence the resorption of bone. In case of BM from DTC, how this process is altered is still unknown [10].

**Fig. 1:**
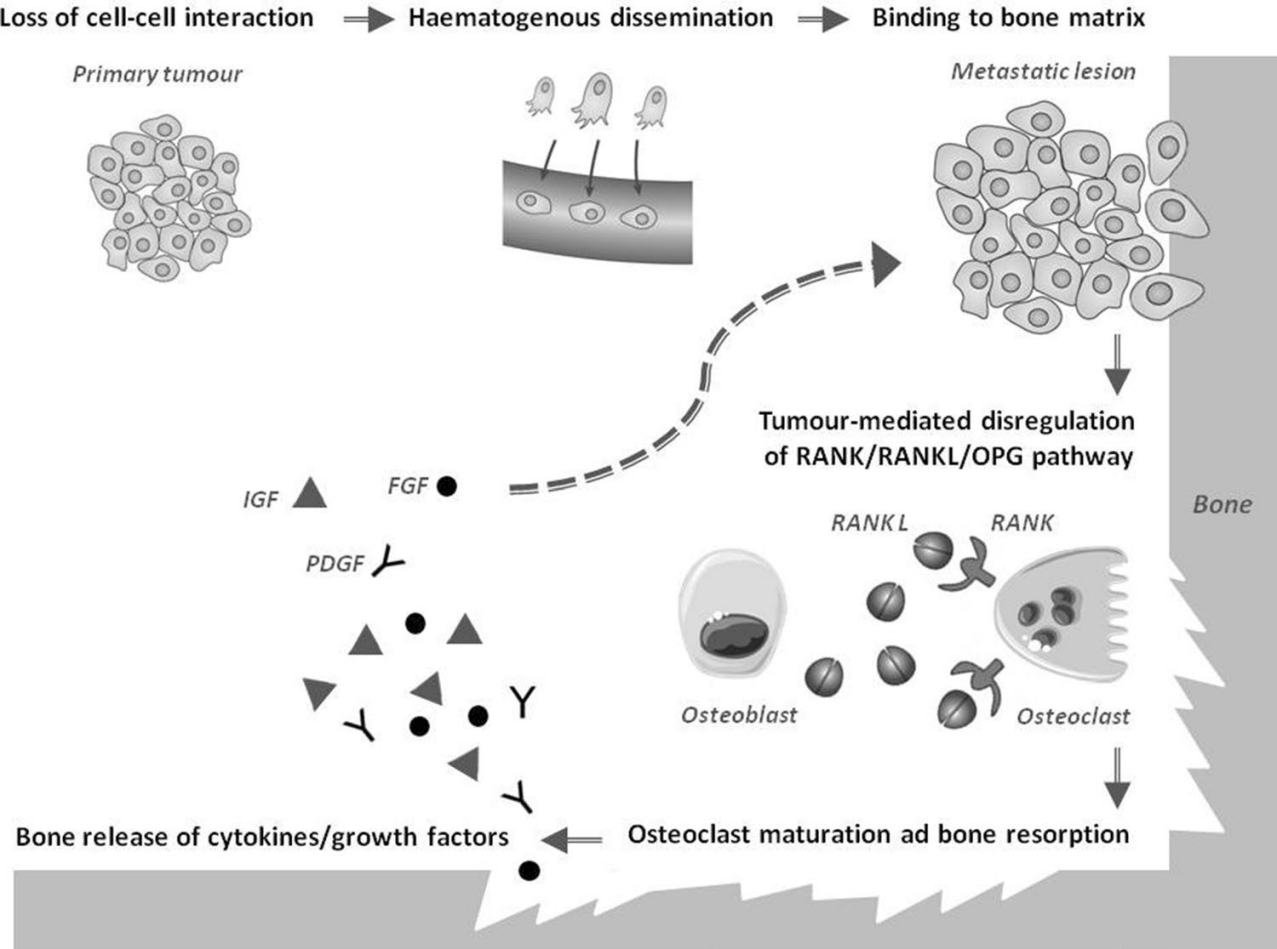
The main mechanisms underlying the development of BM. *BM* bone metastases, *FGF* fibroblast growth factor, *IGF* insulin-like growth factor, *PDGF* platelet-derived growth factor, *RANK* receptor activator of nuclear factor K B, *RANKL* RANK ligand, *OPG* osteoprotegerin

The loss of physiologic cell–cell and cell–matrix interactions is essential for cancer cells to acquire the ability to invade distant sites, including bones. Being a highly vascularised organ, the skeleton is a favourable target for haematogenous invasion: cancer cells are transported by the blood flow and, through specific cell adhesion molecules, are able to bind marrow stromal cells and bone matrix [10]*.* According to the “seed and soil” hypothesis, metastases will develop only in the presence of a favourable microenvironment. Bone is a large repository for growth factors (e.g. insulin-like growth factor, fibroblast growth factor and platelet-derived growth factor), which are released during bone resorption and promote bone homing, colonization, and subsequent tumour growth, as depicted in Fig. 1 [6].

Fibronectin, which normally suppresses cellular migration and adhesion, has been found to be downregulated in FTC cells [10]*.* Conversely, focal adhesion kinase, which promotes tumour invasion, was highly expressed in aggressive thyroid cancers. Interestingly, overexpression of bone sialoprotein and integrin αvβ3 in thyroid cancer cells seems to increase bone adhesion and osteolysis. Also, the different expression of the tumour suppressor genes caveolin-1 and caveolin-2, upregulated in FTC and downregulated in PTC, has been hypothesized to explain the higher propensity of FTC to metastasize to bones when compared to PTC [4]*.* Nevertheless, a whole understanding of the molecular mechanisms involved in the development of BM in DTC is still lacking.

## Prognostic factors

Site, clinical presentation, and burden of metastases are important prognostic factors for DTC, together with age at diagnosis, histological subtype, radioactive iodine (RAI), and ^18^F-fluorodeoxyglucose (^18^F-FDG) avidity [11].

DTC patients with BM generally show a poor prognosis; however, their clinical course may be quite heterogeneous. Asymptomatic patients with RAI-avid BM, but no structural evidence of disease on high-resolution imaging studies, demonstrate excellent responses and better survival [12]. Various retrospective studies tried to identify factors able to independently predict the natural course of DTC patients with BM [13–16]. Nevertheless, many of them lead to inconclusive results, since cohorts were often assembled over multiple decades during which both histopathological evaluation and clinical management have evolved. In several cases, the sample size was very limited to perform meaningful multivariate analyses. Moreover, data about BM were not always separated from those regarding other distant metastases [2].

Nevertheless, at multivariate analysis, the coexistence of non-bone metastases resulted to be an unfavourable prognostic variable in different studies [13, 14], while younger age and extra-spinal BM were found to be independent predictors for improved survival [15]*.* Patients with RAI-avid BM showed a better outcome when compared to subjects with non-RAI avid BM. This finding is somewhat expected, since RAI avidity implies a better differentiation of the tumour itself and a higher efficacy of RAI therapy [3, 13]*.*

A recent study demonstrated that the timing of BM detection could predict survival: a worse prognosis was observed in case of BM detected at the moment of DTC diagnosis, rather than at the moment of the first RAI treatment or during follow-up [16]. A possible explanation of this finding is that patients with BM at diagnosis are likely to have discovered a metastatic DTC following a SRE, while patients with BM detected by RAI therapy often have lesions with no structural correlation. In the same study, the histopathological subtype (PTC or FTC) did not seem to significantly influence the prognosis, even if there was a significantly higher number of patients with FTC in the group with poorer prognosis [16]. In other studies regarding DTC patients with BM, aggressive histotypes (i.e. tall cell, columnar cell, hobnail and insular variants) were associated with a higher risk of disease progression [15], mortality and SREs [17], albeit these findings were not confirmed at multivariate analysis.

## The role of anatomic and functional imaging

### Diagnostic role

**Fig. 2:**
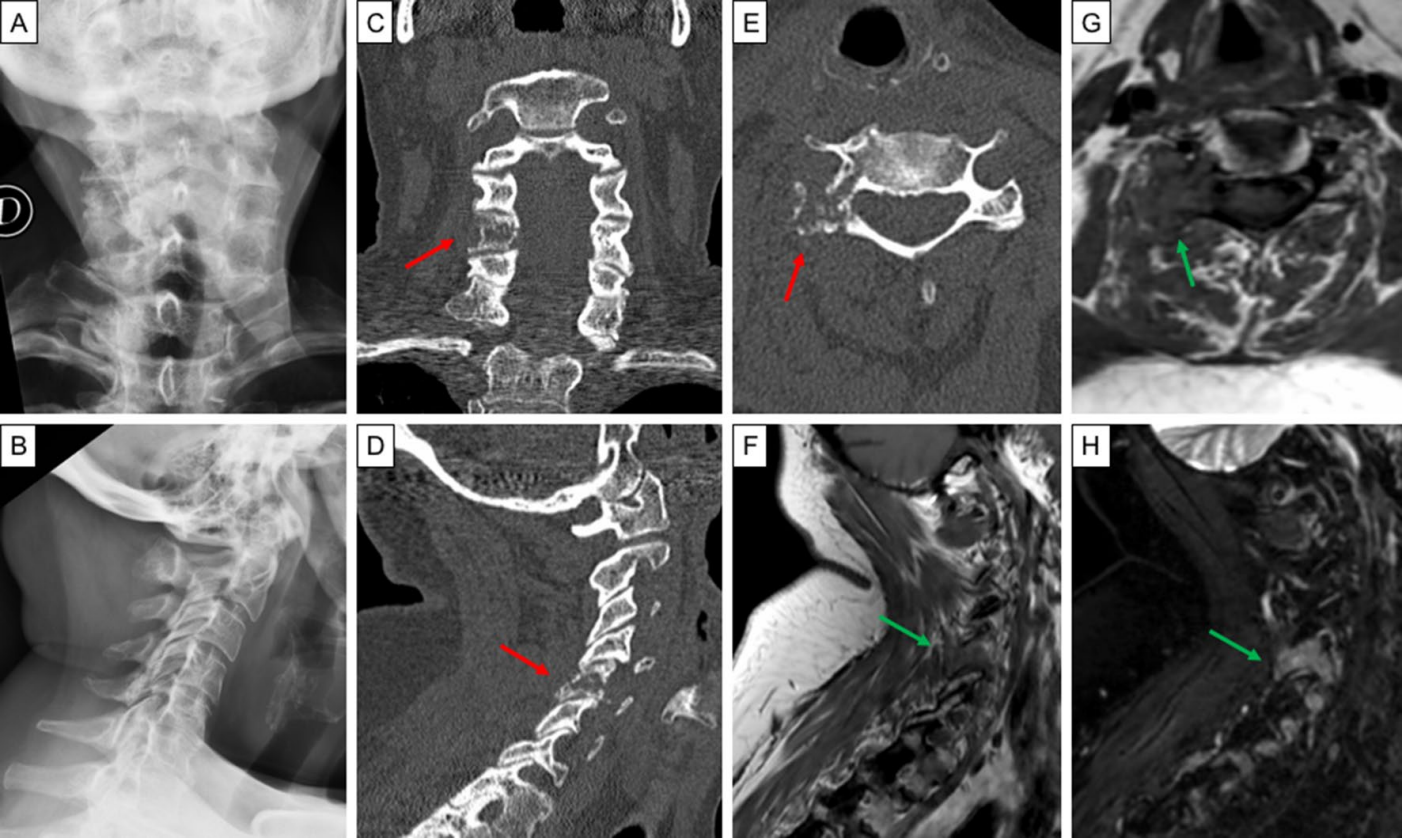
Images from a 55-year-old female with poorly differentiated thyroid cancer and cervical pain. The patient underwent cervical spine plain radiograph (**a** postero-anterior view, **b** latero-lateral view) that was reported as negative. Therefore, after a few days, she underwent cervical spine CT. The CT multiplanar reconstructions images (**c** axial view, **d** coronal view, **e** sagittal view) showed the presence of an osteolytic bone lesion involving the peduncle and the zygapophyseal joint with minimal involvement of the hemisoma of the fifth cervical vertebra (red arrow). The MR images (**f** T1-weighted sagittal view; **g** T1-weighted axial view; **h** T2-weighted with fat suppression sagittal view) confirmed the CT scan findings, with a lesion which resulted hypointense in T1 and hypertense in T2 (green arrow). *CT* computed tomography, *MR* magnetic resonance

BM from DTC are mainly of lytic type and can be frequently associated to an extension into surrounding soft tissues [4]. Plain radiograph is not useful in early detection of small osteolytic lesions, indeed, extensive (30–50%) bone mineral loss is required before it becomes radiographically visible, in particular for the spine and the pelvis. On the other hand, plain radiographs are indicated as first-line imaging study in patients with bone pain or abnormal absorption of radionuclides to exclude a pathological fracture [18]. Computed tomography (CT) and magnetic resonance imaging (MRI) allow to better characterize BM thanks to a higher resolution and a three-dimensional anatomic information (Fig. 2). CT is helpful in visualizing the extension of the lesion and the cortical integrity, also allowing an easy characterization of the lesions as lytic or sclerotic. The sensitivity and specificity reported are 74 and 56%, respectively [19]. Moreover, CT can also be employed for an image-guided bone biopsy. MRI can detect very small BM contributing to an earlier detection of the lesions. The diagnostic accuracy proved to be significantly superior to 16/64-row-slice multidetector CT [20]. Furthermore, in case of vertebral metastases, MRI is essential for the assessment of epidural, nerve, and spinal cord involvement, playing a pivotal role in the pre-surgical workup [21, 22]. Whole-body (WB) MRI with diffusion-weighted imaging (MRI-DWI) is a promising imaging technique in the evaluation of BM from different solid tumours, including DTC [23]. The added value of DWI is the addiction of functional information to morphological sequences: being able to detect differences in cellularity of malignant bone marrow disorders with respect to the normal bone marrow, it might help in differentiating between malignant and benign lesions. WB-MRI-DWI showed a higher sensitivity in the detection of BM from DTC when compared to WB-MRI with standard protocol (82 vs 64%) and similar to ^18^F-FDG positron emission tomography (PET)/CT (79%) [24]. Conversely, in another study involving patients with metastatic DTC, WB-MRI-DWI showed a lower detectability rate (76.5%) for BM comparing to ^18^F-FDG PET/CT (85.7%); both techniques were indicators of poor prognosis [25]. A new MRI perfusion technique (dynamic contrast-enhanced, DCE) has recently been investigated in the diagnostic workup of spinal metastases, showing the ability to detect early vertebral body infiltration and tumour vascularity [26]. The latter characteristic might allow DCE-MRI to become an imaging modality for the evaluation of the response to antiangiogenic therapies. Further investigations need to be carried out, especially in DTC setting, to confirm this hypothesis [27].

Among nuclear medicine functional imaging techniques, WB ^131^iodine (^131^I) scintigraphy is traditionally performed following the administration of RAI therapy: it allows to identify RAI-avid lesions and, in this setting, is more sensitive than ^18^F-FDG PET/CT in the identification of BM [28]. However, planar ^131^I (or ^123^I) WB imaging has limited spatial resolution, which can result in inaccurate localisation and characterization of RAI uptake. By acquiring data in three dimensions, single-photon emission computed tomography (SPECT) imaging enables a more precise differentiation between physiologic uptake and metastatic lesions. The improved accuracy in the characterization of foci of increased tracer uptake emerges especially when functional and structural data are combined in a single imaging session by hybrid imaging (SPECT/CT). In DTC patients with BM, SPECT/CT is superior for the precise localisation and assessment of the extent of bone involvement [29, 30]*.* Moreover, the CT component of SPECT/CT might also detect non-RAI avid lesions [29, 31]*.*

^18^F-FDG PET/CT showed good sensitivity and specificity for the identification of local recurrences or metastases in DTC patients with increased serum Tg levels and negative WB ^131^I scan according to the “flip-flop phenomenon” [32, 33]. The administration of recombinant human TSH (rhTSH) prior to ^18^F-FDG PET/CT imaging in DTC patients with BM did not seem to provide any significant additional information with an increased risk of false positive findings [34]. ^18^F-FDG PET/CT could also provide prognostic information: reduction of OS was observed for PET-positive versus PET-negative BM [28].

Some controversies emerged when comparing ^18^F-FDG PET/CT and ^99m^Technetium-methylene diphosphonate-planar bone scintigraphy (^99m^Tc-MDP-BS) for the detection of BM from different cancer types [35]. In DTC with BM, the accuracy of ^18^F-FDG PET/CT is significantly higher than that of ^99m^Tc-MDP-BS [28, 36]. This is due to ^18^F-FDG PET/CT ability to detect the presence of the tumour directly by its metabolic activity and glucose uptake into cancer cells and, therefore, it might detect BM at an earlier stage [28]. ^99m^Tc-MDP uptake depends on osteoblastic bone reaction to cancer cells, but, as previously described, the lytic nature of BM from DTC limits bone scan accuracy [28].

The role of PET/CT with the bone-seeking radiotracer ^18^fluorine-labeled sodium fluoride (^18^F-NaF) has been studied in few cases of BM from DTC and compared to ^18^F-FDG. ^18^F-FDG PET/CT showed a lower sensitivity in the detection of osteoblastic lesions in comparison to ^18^F-NaF PET/CT, but was more sensitive in evaluating bone marrow involvement and early bone lesions [37–39]. The use of ^18^F-NaF PET in clinical practice should be further evaluated.

The use of ^68^Gallium (^68^Ga) DOTATATE as a radiotracer for PET/CT has also been investigated in RAI-refractory metastatic DTC. However, a study focused on its detection rate of BM from DTC and a direct comparison with other functional imaging techniques is still lacking [40]. Another ^68^Ga-labeled radiotracer (^68^Ga-DOTA-RGD2), an angiogenic marker, has been studied in RAI-refractory DTC patients showing similar accuracy in detecting BM, albeit in a small sized cohort [41].

**Fig. 3:**
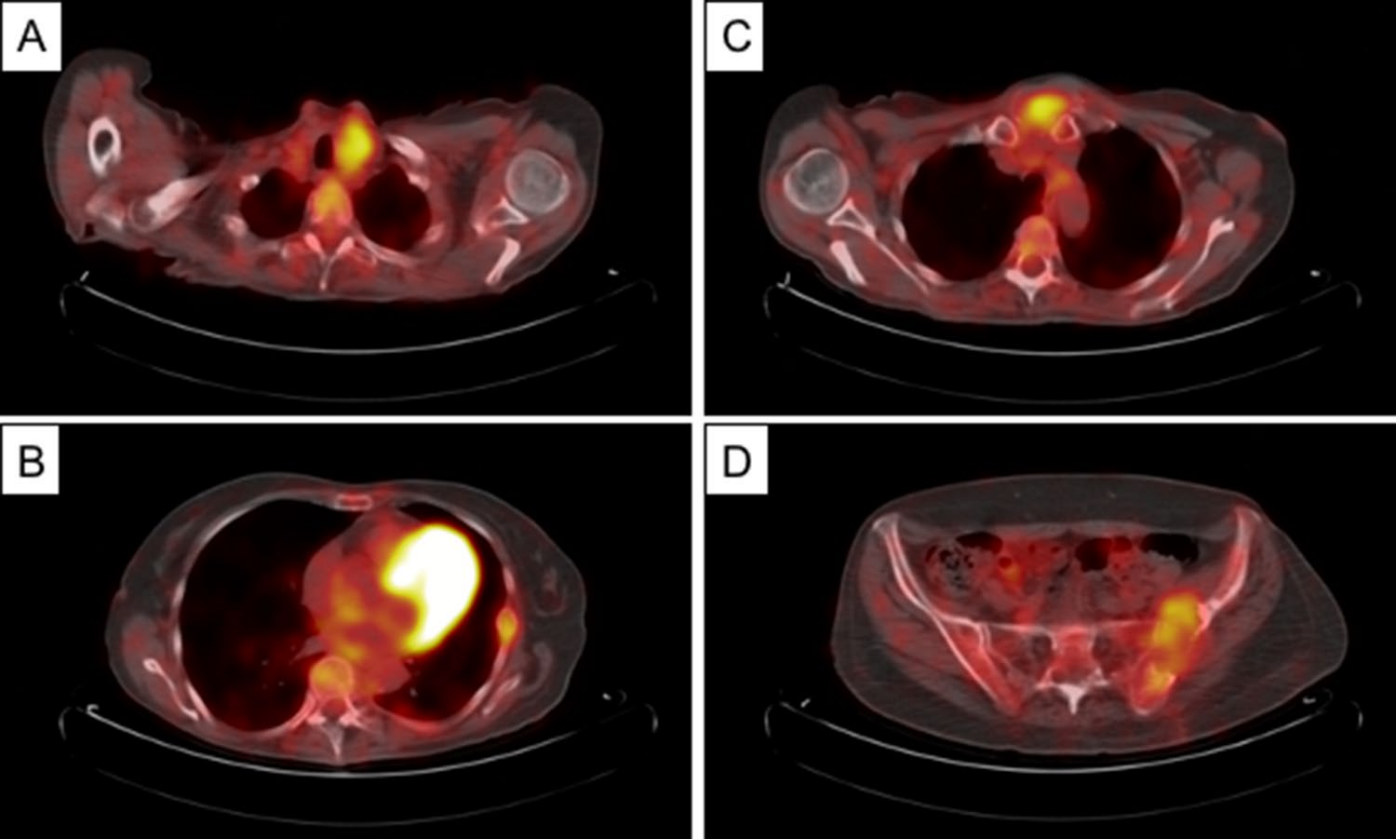
Pre-operatory staging with 18F-FDG PET/CT scan of a 63-year-old female with a lytic lesion of the iliac bone seen at a plain radiograph of the pelvis and subsequently subjected to bone biopsy with histological diagnosis of FTC metastasis. 18F-FDG PET-CT images show the thyroid lesion (**a**) and three bone metastasis involving a rib (**b**), the sternum (**c**) and the iliac wing (**d**). *18F-FDG PET/CT* 18F-fluorodeoxyglucose positron emission tomography/computed tomography, *FTC* follicular thyroid cancer

Compared to anatomical imaging techniques, all functional techniques share the advantage of providing a whole body assessment that is extremely favorable for disease staging (Fig. 3).

The main strengths and limits of the imaging techniques most frequently used in the detection of BM in DTC patients are summarized in Table 1.

**Table 1:** Strengths and limits of the main imaging modalities used for the detection of BM in DTC patients

Imaging technique	Strengths	Limits
Plain radiograph	Diffuse availability Low costs Assessment of potential pathological fractures	Low sensitivity Incapacity of detecting soft tissue involvement
CT	High resolution and three-dimensional information Lesion characterization Assessment of cortical integrity Guidance for bone biopsy	Intermediate sensitivity
MRI	High sensitivity Assessment of soft tissue and neural structures involvement Possibility of employment of functional techniques (DWI, DCE)	High costs Longer scan times Contraindicated in presence of implantable devices
^131^I scintigraphy	High sensitivity for RAI-avid lesions Whole body assessment Theranostic value	Limited spatial resolution (improved by SPECT/CT) Limited value in case of non RAI-avid lesions
Bone scintigraphy	Whole body assessment	Limited spatial resolution (improved by SPECT/CT) Limited accuracy in detecting lytic lesions
^18^F-FDG PET/CT	Whole body assessment High sensitivity in non-RAI-avid lesions Prognostic value	Lower sensitivity than ^131^I scintigraphy for RAI-avid lesions Lower anatomical assessment accuracy than high-resolution CT
^18^F-NaF PET/CT	Whole body assessment Higher sensitivity than ^18^F-FDG PET/CT in detecting osteoblastic lesions	Lower sensitivity than ^18^F-FDG PET/CT in evaluating bone marrow involvement and early bone lesions Low availability Low clinical experience

*CT* computed tomography, *MRI* magnetic resonance imaging, *DWI* diffusion-weighted imaging, *DCE* dynamic contrast enhanced, ^*131*^*I* ^131^iodine, *RAI* radioactive iodine, *SPECT* single-photon emission computed tomography, *PET* positron emission tomography, ^*18*^*F–FDG* ^18^fluorine–fluorodeoxyglucose, ^*18*^*F–NaF* ^18^fluorine–sodium fluoride

### Predictive role

In recent years, an important role of kinase inhibitors (KIs) for the management of RAI-refractory DTC has emerged. The radiological assessment of response to these agents becomes particularly challenging in presence of BM. Response evaluation criteria in solid tumours (RECIST) 1.1 are commonly employed for the evaluation of response to therapy with KIs in cancer patients, including DTC [42, 43]. However, BM without soft tissue involvement measuring ≥ 10 mm—representing the large majority of BM—are designated as unmeasurable, only a frank progression of BM can classify the patient response as unequivocal progression [44].

Bone-specific response criteria were developed at the University of Texas MD Anderson Cancer Center and can be useful in patients with bone-only metastases (MDA criteria). This system allows a greater percentage of BM to be considered measurable disease. Moreover, it takes into account the development of healing sclerosis: visualization of sclerotic lesions or lytic lesions with sclerotic rims might not indicate disease progression (PD) but the healing of previously inconspicuous lesions. Clearly, the so-called osteoblastic flare phenomenon cannot be diagnosed if signs of PD (i.e. appearance or enlargement of other lytic lesions) are detected in other sites [45]. Also bone scintigraphy, as reported in other cancer types such as breast and prostate cancer, can detect flare effect which occurs when healing sclerosis results in an increased bone tracer uptake (or also in the appearance of new osteoblastic lesions), typically within the first 3 months after therapy. In this case, the combination of clinical information, the use of other imaging studies (CT/MRI), and the evolution on sequential imaging are essential for a correct interpretation of the scan findings [39]. However, no focused evaluation of the BM response to systemic therapy in DTC patients according to these criteria is currently available, to our knowledge.

Being cytostatic drugs, KIs might not always determine a profound change in tumour size despite their effectiveness. Therefore, morphologic-based criteria could not detect the actual tumour response and fail to demonstrate the real clinical benefit. ^18^F-FDG PET/CT scan can be a useful method to measure metabolic response of ^18^F-FDG-avid BM [45]. Positron emission tomography response criteria in solid tumours (PERCIST) have been proposed to measure disease response through the assessment of metabolic activity variation. Although they are employed in clinical trials, their use in real-life practice is not usual [46].

Recently, early metabolic assessment in apatinib-treated RAI-refractory DTC has been investigated [47]. However, the role of functional imaging and its correct timing need to be further explored along with anatomic criteria for the evaluation of BM changes during systemic treatments in DTC patients.

## Management

The main recommendations regarding the management of BM in DTC included in the most recent guidelines are summarized in Table 2 [32, 48–50].

**Table 2:** Recommendations of the American Thyroid Association (ATA) guidelines (2015), Italian Consensus (2018), National Comprehensive Cancer Network (NCCN) (2019), and European Thyroid Association (ETA) Guidelines (2019) regarding the management of BM in DTC

Active surveillance^a^	Local treatments	Systemic treatments
ATA guidelines (2015) [32]		
Serial controls (3–12 months) In asymptomatic, stable or minimally progressive RAI-refractory disease, with low probability of complications	In case of single or few threatening and/or symptomatic BM. Before or during systemic therapy: • Surgery • RT Alone (for pain relief or palliation) or complementary to surgery (in case of incomplete resection). SBRT (different protocols, maximum 30 Gy) preferable for higher efficacy and limited radiation to the spinal cord • Percutaneous procedures RFA or cryoablation, for rapid and long-lasting pain control; cryoablation can treat larger BM than RFA; frequently associated with cementoplasty (promising in purely lytic BM)	• RAI therapy RAI activity: 100–200 mCi or determined by dosimetry. In case of iodine-avid BM • KIs Approved as first line: sorafenib or lenvatinib*.* Alone or in combination with local treatments. In rapidly progressive, symptomatic and/or threatening RAI-refractory disease, not otherwise amenable to local control • Bone-directed agents. Bisphosphonates, especially zoledronate, and denosumab. Alone or concomitantly with other systemic/local therapies. For delaying occurrence of SREs and improving symptoms, in case of diffuse and/or symptomatic BM
Italian Consensus (2018) [48]		
Controls at regular intervals (3–12 months) In asymptomatic, stable or slowly progressive RAI-refractory metastatic disease, without life-threatening lesions	Strongly suggested whenever progression of the disease or its riskiness are related to a single lesion: • Surgery • RT • Percutaneous procedures	• RAI therapy RAI activity: 100–200 mCi or determined by dosimetry. In case of iodine-avid BM • KI In case of RAI-refractory disease, rapidly progressive, significantly symptomatic and/or with life-threatening lesions not suitable for local therapies • Bone-directed agents Not mentioned
NCCN guidelines (2019) [49]		
Periodical controls In asymptomatic and indolent RAI-refractory disease	In case of symptomatic lesions or asymptomatic but in weight-bearing sites: • Surgery • RT • Percutaneous procedures Consider embolization prior to surgical resection to reduce the risk of hemorrhage	• RAI therapy RAI activity: 100–200 mCi or adjusted by dosimetry. In case of known/suspected distant iodine‐avid BM. Consider alternative therapies before RAI administration to prevent invasion/compression of vital structures or pathologic fracture (as a result of TSH stimulation) • KIs Approved as first line*:* sorafenib or lenvatinib (preferable). For progressive and/or symptomatic RAI-refractory disease • Bone directed agents Intravenous bisphosphonates (e.g. pamidronate or zoledronate) or denosumab in RAI-refractory BM to prevent SREs
ETA guidelines (2019) [50]		
Serial controls (4–6 months) In case of slow growth (< 20% in 12–14 months)	In case of progression of a single lesion or more than one lesion within the same organ. Before or during systemic therapy • Surgery • RT For local disease control and pain relief • Percutaneous procedures RFA employed in case of no surgical indication or prior to surgery, to reduce the volume of a lesion. Cementoplasty (alone or in combination with RFA or RT) for lytic BM to prevent pathological fractures and to reduce pain	• RAI therapy Not mentioned^b^ • KIs Approved as first line*:* sorafenib or lenvatinib. Alone or in combination with local treatments. In progressive RAI-refractory disease with considerable tumour load and potential clinical complications without systemic treatment • Bone-directed agents Especially zoledronate and denosumab

*BM* bone metastases, *Gy* gray, *mCi* milliCurie, *RAI* radioactive iodine, *RT* radiotherapy, *SBRT* stereotactic body radiotherapy; *RFA* radiofrequency, *SREs* skeletal-related events, *KIs* kinase inhibitors

^a^Under TSH suppressive thyroid hormone therapy

^b^These guidelines specifically regards RAI-refractory disease

### Radioiodine therapy

In presence of RAI-avid metastases, RAI therapy is normally used as a first-line treatment in DTC patients [32]. However, although RAI may eradicate small metastases, it is poorly effective in treating larger lesions [12]. Moreover, metastatic patients might show complete or partial lack of RAI uptake, with a significant negative impact on prognosis [50].

In the specific setting of BM, RAI therapy was found to exert favourable effects on survival in patients with RAI-avid BM [17] and, as previously underlined, these patients show better survival rates when compared to patients with non-RAI avid BM [13]. According to a recent retrospective study, RAI treatment in combination with one or more non-RAI local or systemic treatments was associated with a significantly increased OS compared with RAI therapy alone [51]. It has also been reported that RAI therapy reduced pain originating from BM [52].

However, when compared to other metastatic sites, RAI therapy resulted less effective in the treatment of BM: for instance, patients with lung metastases showed higher remission rates (50–74%) than patients with BM (10–17%) [3, 53], moreover, more than 20% of BM do not show any RAI uptake [3, 54].

The efficacy of RAI therapy on BM and its impact on progression-free survival (PFS) and OS are related to several factors. The cumulative activity of RAI resulted significantly associated with improved survival [54]. International guidelines recommend high and repeated activity of at least 3.7–7.4 GBq for lung and bone disease, respectively. However, the potential harms of repeated doses of RAI (i.e. bone marrow suppression) should be carefully taken into account and dosimetry, in this setting, can be a useful tool to reduce the risk of long-term toxicity [32].

Dosimetric studies allow to evaluate the absorbed dose to each lesion. The dosimetric approach demonstrated higher efficacy when compared to the empiric approach in locally advanced DTC [55]. Conversely, in the context of metastatic disease, no significant differences were found in terms of OS and PFS comparing empiric versus dosimetric approach, especially in patients with multiple and larger metastases [55–58]. In the specific setting of DTC patients with BM, no controlled studies compared the benefit of empirical RAI administration to a dosimetrically determined dose of RAI therapy.

Both ^131^I or ^124^I isotopes can be used to quantify RAI uptake; for this purpose, the use of ^124^I PET/CT seems to be extremely helpful thanks to both the PET images and the longer half-life of ^124^I [59, 60]. Jentzen et al*.* reported a low efficacy of RAI therapy for BM even using a dosimetry-guided approach with ^124^I PET/CT; it was also confirmed the need of higher absorbed doses for BM to obtain a response compared to those reached for lung metastases [61]. However, the use of ^124^I is hampered by its restricted availability.

**Fig. 4:**
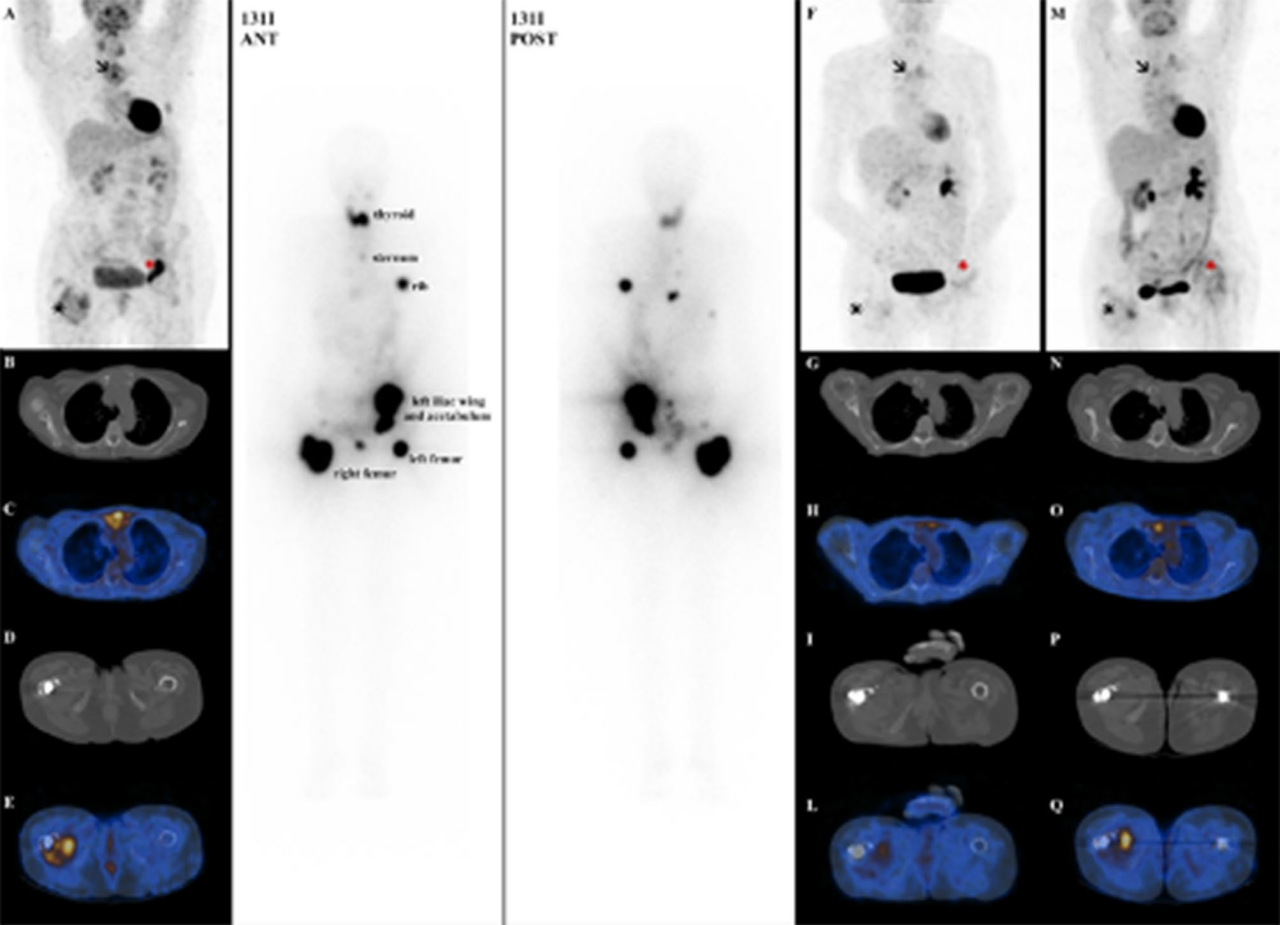
Case presentation of a 67-year-old female patient with metastatic PTC. (**a**–**e**) images show baseline 18F-FDG PET/CT scans. After the diagnosis, she underwent total thyroidectomy (pT3Nx) with a partial resection of the sternum metastasis, and RAI treatment (activity 5.5 GBq). The 131I whole-body scan (131I ANT and 131I POST) showed RAI uptake in all metastases detected at the baseline 18F-FDG PET/CT, with an heterogenous FDG-avidity (131I+/18F-FDG+). A second RAI treatment was not performed for persistent neutropenia; for this reason, the patient was candidate for locoregional therapy (RFA of the left iliac wing and left acetabulum) and she was followed-up with 18F-FDG PET/CT. The first follow-up 18F-FDG PET/CT scan (**f**–**l**) demonstrated a very good metabolic partial response of the left iliac wing and left acetabulum BM (red asterisk), with also a decreased tracer uptake at the right femur level (black asterisk). Despite that, 18F-FDG PET/CT scan performed after 12 months of follow-up (**m**–**q**) showed disease progression, with the appearance of new increased tracer uptake in all the previous sites of disease (sternum, left iliac wing and left acetabulum and right femur). *PTC* papillary thyroid cancer, *18F-FDG PET/CT* 18F-fluorodeoxyglucose positron emission tomography/computed tomography, *RAI* radioactive iodine, *RFA* radiofrequency ablation, *BM* bone metastases

RAI refractoriness is defined according to five different scenarios: (1) no RAI uptake is present on a diagnostic ^131^I WB scan; (2) no RAI uptake is present on a ^131^I WB scan performed several days after RAI therapy; (3) RAI uptake is not present in all tumour foci but only in some of them; (4) disease progression despite RAI uptake; (5) disease progression despite a cumulative RAI activity > 22.2 GBq (600 mCi) [62]. In metastatic patients, ^18^F-FDG PET/CT should be used as a complementary tool to RAI WB scan to predict BM response to RAI and to better define RAI-refractory DTC. Patients with ^18^FDG-avid and non RAI-avid distant metastases generally have a rapidly progressive disease. In contrast, patients with RAI-avid and ^18^F-FDG negative lesions have a better prognosis. Patients with both ^18^F-FDG and RAI uptake in the same lesion or in different lesions represent a very heterogeneous group, but their prognosis seems to be similar to the group with only ^18^F-FDG uptake [63] Therefore, ^18^F-FDG PET/CT could help to identify tumours or single lesions with an aggressive behavior that could benefit from other local or systemic therapies different from RAI (Fig. 4) [62].

It is worth remembering that RAI treatment may be contraindicated for large BM at certain sites (e.g. cranium or spine), due to the possible enlargement of the tumour lesions induced by TSH increase either obtained by the administration of rhTSH or after hormone withdrawal, which can lead to compressive symptoms [64].

### Surgery

Surgery is a possible treatment option for BM, especially for spinal metastasis. Surgical approach is mainly indicated in presence of impending fracture risk, persistent pain, and spinal instability (with or without spinal compression and neurologic injury) [4].

Albeit not always feasible, some authors suggested that complete resection of macroscopically identified bone tumour should be attempted, since this strategy has been associated in some studies with better OS, especially in younger patients with single or few BM. The survival advantage seems to be evident for both appendicular and spinal BM [54, 65–67].

In contrast, other studies did not find any significant reduction in overall mortality in surgically treated patients [17, 68]. Nevertheless, total en-bloc spondilectomy (TES) has been associated with lower risk of local recurrence and need for reintervention [68].

When aggressive surgical resection is not feasible (i.e. extensive metastatic disease, comorbidity, advanced age, size of BM), despite the lack of a survival benefit, surgery remains a valuable choice for the palliation of BM-related symptoms or the prevention of pathological fracture and spinal cord compression in case of BM in weight-bearing sites [69].

### Radiotherapy

External beam radiation therapy (EBRT) is widely used in clinical practice. It can complement surgery as adjuvant treatment or be used alone, in case of refractory bone pain, for prevention of pathological fractures or in case of spinal compression [70]. Despite the relative radioresistance of DTC [71], EBRT represents an effective and safe treatment approach for non-surgical candidates with symptomatic BM or asymptomatic BM at higher risk of fracture and/or neurological symptoms.

Generally, the onset of the therapeutic effect is not immediate after the end of the radiotherapy course, unless a single dose of 8 Gy is used. More typical fractionation schemes, however, consist of 20 Gy in five fractions in 1 week, or 30 Gy in ten fractions in 2 weeks [72, 73].

The development of a more accurate technique called stereotactic ablative radiotherapy or stereotactic body radiotherapy (SABR or SBRT) has changed the treatment paradigm, especially for spinal metastases. It achieves a high level of conformality around the target with a steep dose fall-off, providing higher radiation doses (14–16 Gy in a single fraction) to the target lesions and limiting the dose and the risk of damage to the surrounding critical organs [74]. Thanks to these characteristics, SABR could achieve better results and, if necessary, it allows the reirradiation of previously treated sites.

Few studies investigated the efficacy of SABR in DTC patients with BM; furthermore, SABR protocols differed among them [75–78].

A prospective evaluation of frame-based SABR was performed in thyroid cancer patients (mainly DTC) treated with intensity-modulated radiation therapy (IMRT) in single or multi-fraction schedules (from 16–18 Gy in one fraction to 27–30 Gy in three to five fractions) as primary or adjuvant/salvage therapy. Local control rates were 88% at 2 years and 79% at 3 years, but pain flare was reported by 30% of patients [75].

**Fig. 5:**
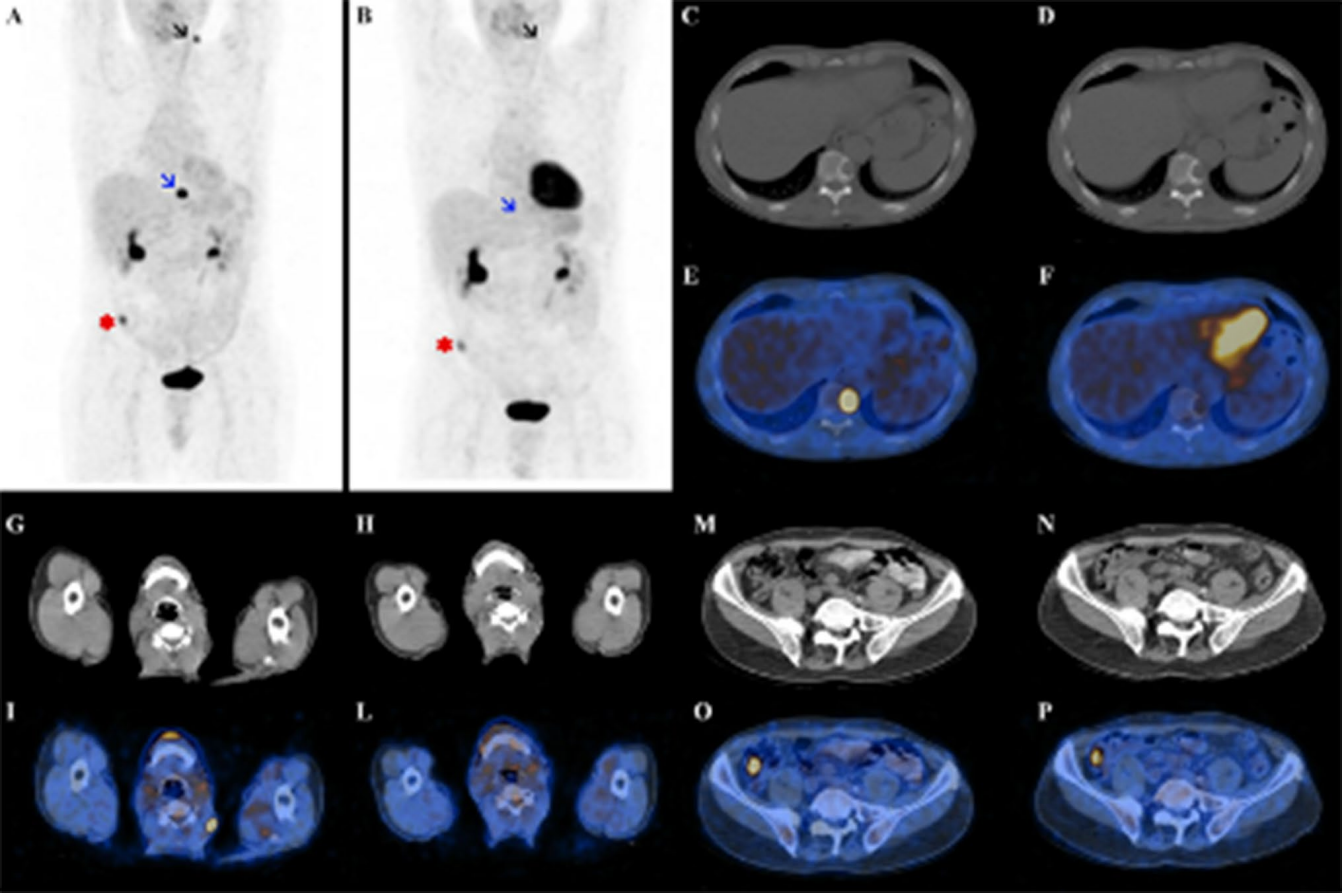
Case presentation of a 68-year-old male patient with metastatic PTC. He underwent total thyroidectomy (pT3mN1bMx) and two consecutive RAI treatments (cumulative activity 11.1 GBq). After the second RAI treatment, 18F-FDG PET/CT demonstrated three FDG-avid lesions: a left cervical lymph node (black arrow on **a** image; see also **g** and **i**), a lytic bone lesion in D10 (blue arrow on **a** image; see also **c** and **e**) and a focal intestinal uptake (asterisk on **a** image; see also **m** and **o**). So, the patient underwent a cervical lymphadenectomy that confirmed the thyroid origin of the lesion, and a Cyberknife radiosurgery of the D10 bone lesion (24 Gy). The subsequent follow-up 18F-FDG PET/CT scan demonstrated a complete metabolic response of the D10 bone lesion (blue arrow on **b** image; see also **f**), even if its radiological aspect was unchanged on CT (**d**); moreover, no further pathological radiotracer uptake was detected in the left cervical region (black arrow on **b** image; see also **h** and **l**). The focal intestinal uptake was stable (asterisk on **b** image; see also **n** and **p**) and was diagnosed as an adenomatous polyp at the subsequent polypectomy. The 18F-FDG PET/CT findings were further validated by the concomitant reduction of serum Tg levels. *PTC* papillary thyroid cancer, *RAI* radioactive iodine, *18F-FDG PET/CT* 18F-fluorodeoxyglucose positron emission tomography/computed tomography, *Tg* thyroglobulin

A retrospective study showed a 1-year local control rate of 97.5% and a significant improvement in reported symptoms in a small series of 13 DTC patients with a total of 60 BM (both spinal and non-spinal) treated with Cyberknife (1–10 fractions, with a median dose of 27 Gy), a dedicated radiosurgical system consisting of a robotic arm with a linear accelerator and a target tracking system. Most irradiated lesions tended to shrink or decrease in growth rate after SABR [76]. Another retrospective study showed promising results with this technique [77]. Figure 5 is an example of a successful Cyberknife treatment. Conversely, lower rates of local control (67% at 1 year) were observed in a recent retrospective cohort of 12 patients with a total of 32 spinal metastases treated with Cyberknife [78]. These findings may be mainly explained by the baseline patient characteristics (high rate of extra-spinal metastases, poor performance status [PS]).

In all the previous cited studies, SABR was generally well tolerated and not associated to the onset of myelopathy. However, a significant risk of vertebral compression fractures has been described after this type of treatment, particularly in older subjects, in case of a pre-existing fracture or osteolytic lesions with high tumour burden, high radiation dose and baseline pain. Patients with these risk factors should be carefully evaluated for prophylactic stabilization prior to SABR to avoid this potential complication [79, 80].

Although the use of SABR seems to lead to a significant improvement in PFS and OS in patients with oligometastatic disease from other types of cancer [81], the real impact on the survival rate of DTC patients remains to be established. A recent real-life retrospective evaluation did not show any significant effect of EBRT in reducing the overall mortality of DTC with BM at multivariate analysis [17]. Further studies are needed to select those patients amenable to sophisticated radiotherapy techniques such as SABR.

### Percutaneous procedures

Interventional radiology plays an important role in the management of single or few BM from DTC. It can be a less aggressive alternative to surgery in selected patients (i.e. in case of poor patient PS, or local recurrence at the site of previous surgery), both before or during systemic therapy in case of symptomatic BM at higher risk of local complications. Although patients are usually referred for percutaneous procedures due to their symptomatic status, a more relevant curative role has been hypothesized and need to be further investigated [82].

The available percutaneous techniques can be divided in vascular, ablative and consolidative treatments, which could be applied alone as well as in combination [83]. Published experience in DTC patients is limited and randomized prospective studies comparing the efficacy and tolerability of different procedures are lacking. Their choice in clinical practice is mainly based on local experience, lesion site, and patient preference [32].

Embolization techniques: embolization therapy aims to achieve an extended devascularisation of the tumour tissue through vascular occlusion with different agents, causing ischaemia, and subsequent necrosis. The rational of its use in case of BM from DTC is that these lesions are usually hyper-vascularised. In selected patients, embolization therapy has been employed in DTC with BM either alone or in combination with other treatment modalities [83].

This treatment does not seem to improve life expectancy, but may achieve remission of symptoms and reduction of tumour burden [84]. The embolization procedure could facilitate subsequent surgical interventions by inducing tumour shrinkage, reducing intraoperative bleeding and allowing a better delimitation between the tumour and the surrounding tissues [85, 86].

Better results in terms of duration of symptoms control without tumour progression were observed when this treatment was combined with EBRT or RAI therapy [87]*.*

Ablation techniques: radiofrequency ablation (RFA) and cryoablation (CA) can achieve necrosis of the tumour tissue by an increase or a decrease of intra-tumour temperature, respectively. Their use has been described also in the context of DTC with BM [82, 88]. Another form of thermal ablation is microwave ablation (MWA), which uses electromagnetic waves to increase the intra-tumour temperature. When all these techniques are applied to sites exposed to mechanical stresses, a subsequent consolidation with surgical or percutaneous procedures can avoid secondary bone fractures [83].

Ablation techniques have potential advantages compared with surgery and radiation therapy, including reduced morbidity, repeatability, lower procedural cost, short procedural time and the possibility to be performed in an outpatient setting [89]; moreover, they were found to be, either alone or in combination with cementoplasty, effective and safe treatments for painful metastases [87].

Cementoplasty: percutaneous cementoplasty is a minimally invasive procedure which consists in a polymethylmethacrylate (PMMA, also referred to as “bone cement”) injection into bone segments with structural weakness, obtaining biomechanical stability and pain relief [90, 91]. The best candidates for this procedure are patients suffering from refractory pain due to osteolytic spinal metastases, conversely, cementoplasty is not indicated in purely osteoblastic lesions that, anyway, are rare in DTC. Available studies regarding vertebroplasty in DTC are mostly small series or case reports [91]. Cazzato et al. reported a single-institution experience in percutaneous image-guided treatment of BM from DTC; cementoplasty was performed in 77.5% of BM and was associated with a good percentage of local tumour control [82]. Vertebroplasty can be combined with other procedures, such as RFA and RAI therapy [92, 93].

### Kinase inhibitors

In recent years, KIs have been successfully employed for the treatment of progressive RAI-refractory DTC with distant metastases, included BM [43]. According to the most recent guidelines, systemic treatment should be considered for patients with progressive RAI-refractory disease and considerable tumour burden [32, 48–50].

In a post hoc analysis of a phase III trial, patients with BM treated with lenvatinib showed a PFS benefit in comparison with their non-treated counterpart (median PFS 14.8 vs 2.1 months, HR 0.26) [94].

Conversely, a worse response to treatment and a shorter PFS have been reported in different studies that included patients treated with sorafenib and sunitinib in case of BM [95–98]. Interestingly, BM which had received EBRT before the onset of KI therapy were found to be stable during KI therapy, while non-irradiated BM experienced progression despite a response to KI being observed in extra-bone lesions [95]. Progression of BM while on KI might occur despite maintained benefit at other metastatic sites [99]. These findings suggest the need of a multimodal approach for the management of DTC patients with BM also after initiation of cytostatic therapy, which should be continued with the aim of optimizing systemic disease control.

### Antiresorptive treatments

Acting as potent inhibitors of bone resorption by inducing apoptosis of osteoclasts, bisphosphonates (BPs) have been widely used for preventing or delaying SREs, mainly in patients with breast and prostate cancer [100, 101]*.* More recently, denosumab, a monoclonal antibody that inhibits osteoclast activity by targeting the RANK ligand, has been successfully employed in these patients, showing a stronger efficacy in delaying SREs when compared to zoledronic acid (ZA) [102]*.*

The effects of bone-directed therapy in DTC patients with BM have been investigated in a limited number of patients only. In ten DTC patients with BM, administration of pamidronate was associated with reduction of bone pain and improved quality of life [103]. In retrospective evaluations of DTC patients with BM, SREs incidence was significantly lower in ZA-treated patients than in non-treated patients [104, 105]. The use of ZA significantly delayed the onset of the first SRE [104]*.* A prospective study enrolled 19 DTC patients with BM to receive ZA every 4–5 weeks: a minor occurrence of metastatic spinal cord compression was observed in these patients when compared to 16 non-treated historical controls [106]. More recently, a study investigated the outcome of DTC patients with BM who received only RAI therapy with those who were also treated with non-RAI therapies. In this population, patients who received denosumab showed a better survival than those who did not, even subjects treated with BPs seemed to have slightly improved survival, but statistical significance was not reached [51].

Unexpectedly, the use of BPs or denosumab in DTC patients with BM is still limited in clinical practice. A retrospectively evaluation of the real-life management of BM in 143 patients with DTC revealed that only 22.4% of them received anti-resorptive bone-active therapy (ZA in all but one cases), mainly in case of pre-existing SREs [17].

Adverse events (AEs) associated with antiresorptive therapy are generally mild and can be easily managed. During intravenous BPs, acute phase reaction (a flu-like syndrome characterized by fever and arthralgias/myalgias) mainly occurs after the first administration and it is usually self-limiting, while BPs-induced nephrotoxicity is directly related to dose and infusion time [65]. With denosumab, fewer renal AEs and acute-phase responses have been reported, while hypocalcemia seems to occur more frequently. In patients treated with antiresorptive treatment, an adequate vitamin D and calcium supplementation are essential to prevent hypocalcemia and reach an optimal bone mineral density response, especially in case of hypoparathyroidism. Osteonecrosis of the jaw (ONJ) and atypical femoral fractures are rare, but serious AEs of antiresorptive treatments, the incidence rates of these side effects with ZA, and denosumab do not differ significantly [107, 108]*.* Since the risk of ONJ is higher in patients with malignancies and during chemotherapy or head and neck EBRT, a careful dental evaluation prior to initiation of antiresorptive treatments is mandatory in these patients [65].

The potential harms and benefits of concomitant use of antiresorptive treatments and KIs remain to be established, since anti-angiogenic therapies have been associated with the occurrence of ONJ also in absence of antiresorptive treatments in DTC [109]. A study investigating the efficacy of lenvatinib combined with denosumab in the treatment of patients with predominant bone metastatic RAI refractory DTC (LENVOS) is ongoing (NCT03732495). Recently, a retrospective study included 23 patients with BM from thyroid cancer (mainly DTC) treated with denosumab, mostly in association with KI therapy: ONJ and severe hypocalcemia occurred in 26% and 13% of patients, respectively [110]*.*

The optimal dosing interval for bone-directed therapy is still uncertain. Among breast cancer patients, ZA therapy every 12 week—rather than every 4 week—has been proposed and might be considered an acceptable treatment schedule. Indeed, no difference in terms of SREs occurrence has been observed, while incidence of ONJ and kidney dysfunction resulted lower in the 12-week population, even if statistical significance was not reached [111]*.* In DTC population, randomized trial data to clarify this issue have not yet been published. Also, the ideal duration of antiresorptive therapy in this population remains controversial.

## Conclusion

In patients with metastatic DTC, BM can be frequently detected and are generally associated with low survival rates. SREs might represent a serious complication of BM, resulting in high morbidity and impaired quality of life. A whole understanding of the molecular mechanisms involved in the development of BM in DTC is still lacking. Several factors were found to predict the natural history of the disease in patients with BM (e.g. age, RAI-avidity, lesion size, time of diagnosis, coexistence of non-bone metastases). A careful multidisciplinary and personalized evaluation is essential to improve the clinical outcome of each patient. BM from DTC can be detected by anatomic imaging (CT and/or MRI) or functional assessment (mainly ^131^I scintigraphy with the added value of SPECT/CT and/or ^18^F-FDG PET/CT). RAI therapy exerts favourable effects on patient survival in case of RAI-avid BM, but it is not curative in case of high volume BM and in case of RAI-refractory disease. Local treatments (e.g. surgery, radiotherapy including SABR, and percutaneous procedures such as embolization, radiofrequency ablation, cryoablation and cementoplasty) showed good results, alone or in combination with systemic treatment. More recently, KIs have been introduced for the management of patients with progressive RAI-refractory disease and considerable tumour burden, including subjects with BM. The radiological evaluation of response to systemic therapy is challenging: RECIST criteria have several limits in presence of BM. The effects of bone-directed therapy (BPs and denosumab) have been investigated in a limited number of patients only. Future prospective studies focusing on still unsolved issues (e.g. best RAI approach, dose and schedule of BPs, potential role of immunotherapy, optimal sequence of BM treatments, novel techniques of treatment, etc.) are needed to personalize the management of these patients and improve their clinical outcome.
